# Supplementary material for: Presynaptic GABAA receptors control integration of nicotinic input onto dopaminergic axons in the striatum
Source: bioRxiv. 2024 Jun 25:2024.06.25.600616. Preprint. [Version 1] doi: 10.1101/2024.06.25.600616 (PMC11451734; doi:10.1101/2024.06.25.600616)
Supplement: Supplement 1 [file NIHPP2024.06.25.600616v1-supplement-1.pdf]

bioRxiv preprint doi: https://doi.org/10.1101/2024.06.25.600616; this version posted June 25, 2024. The copyright holder for this preprint (which was not certified by peer review) is the author/funder. This article is a US Government work. It is not subject to copyright under 17 USC 105 and is also made available for use under a CC0 license.

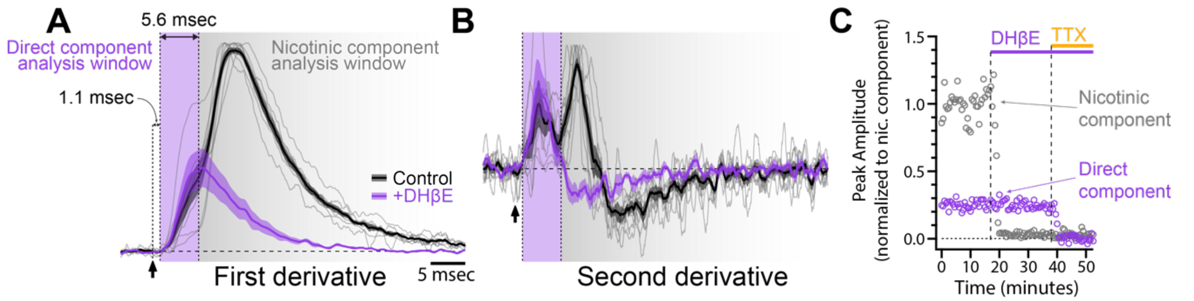

Supplemental Figure 1: Definition of direct and nicotinic components in jGCaMP8s photometry traces. (A) First derivative of evoked jGCaMP8s signals normalized to the peak during control trials. Thin lines represent data from 8 individual experiments. The black trace is the average of all experiments during the control condition, and the purple trace is the average of the same experiments following application of DHβE (1 μM). Analysis windows are defined based on traces in (B). **(B)** Second derivative of the traces shown in (A). Direct component analysis window (purple region) begins at the onset of the first peak and ends at the onset of the second peak, when the nicotinic component analysis window (grey region) begins. **(C)** Time course of the direct and nicotinic component for the slice shown in Fig. 2B and E during DHβE and TTX wash-in.

bioRxiv preprint doi: https://doi.org/10.1101/2024.06.25.600616; this version posted June 25, 2024. The copyright holder for this preprint (which was not certified by peer review) is the author/funder. This article is a US Government work. It is not subject to copyright under 17 USC 105 and is also made available for use under a CC0 license.

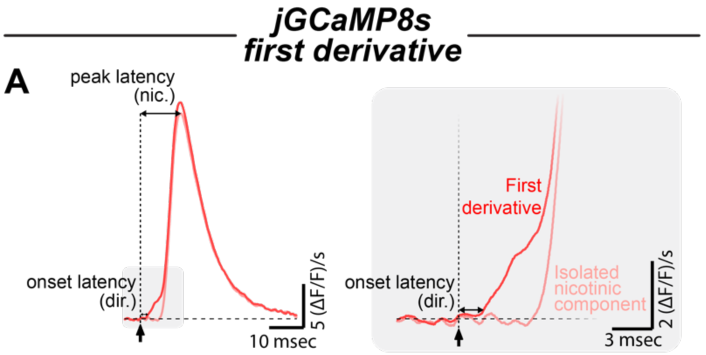

Supplemental Figure 2: Explanation of latency measurements for direct and nicotinic components. (A) Illustration of where measurements were made for Fig. 3G. Peak latency of the nicotinic component was defined as the time at which the subtracted trace reached its maximum value. Onset latency of the direct component was defined as the time at which the first derivative trace crossed 10% of the direct component peak amplitude. Onset latency was used for the direct component because the peak of the component did not always fall within the boundaries of the analysis window.

bioRxiv preprint doi: https://doi.org/10.1101/2024.06.25.600616; this version posted June 25, 2024. The copyright holder for this preprint (which was not certified by peer review) is the author/funder. This article is a US Government work. It is not subject to copyright under 17 USC 105 and is also made available for use under a CC0 license.

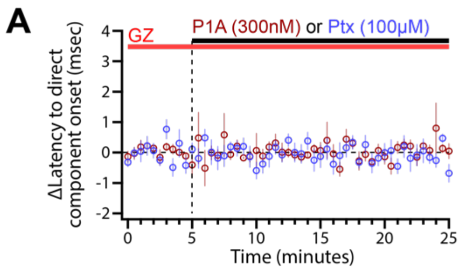

Supplemental Figure 3: Direct component onset latency is not impacted by P1A or picrotoxin. **(A)** Time course of the onset latency of the direct component following application of either picrotoxin (n = 8) or P1A (n = 5). All slices were bathed in ACSF containing 10 μM gabazine for the duration of the experiment. From the same experiments as Fig. 6G.
